# Correlation of internal jugular and subclavian vein diameter variation on bedside ultrasound with invasive right heart catheterization

**DOI:** 10.1016/j.ihj.2021.01.024

**Published:** 2021-02-02

**Authors:** Gaurang Nandkishor Vaidya, Shahab Ghafghazi

**Affiliations:** Department Cardiovascular Medicine, University of Louisville, Louisville, KY, USA

**Keywords:** Bedside ultrasound, Internal jugular vein, Subclavian vein, Fluid overload, Heart failure, Imaging

## Abstract

**Introduction:**

Accurate estimation of fluid status is paramount in patients with heart failure. We hypothesized that bedside ultrasound assessment of the internal jugular vein (IJV) and subclavian vein (SCV) could reliably estimate right atrial pressure (RAP).

**Methods:**

Prospectively enrolled patients were positioned supine. IJV was imaged at the apex of the right sternocleidomastoid muscle and SCV was imaged at the lateral third of the right clavicle. Using M-mode on a portable ultrasound machine, the maximum (D_max_) and minimum (D_min_) anteroposterior diameters were noted during normal breathing. Respiratory variation in diameter (RVD) was calculated as [(D_max_ – D_min_)/D_max_] and expressed as percent. Collapsibility was assessed with sniff maneuver. Patients then underwent right heart catheterization and their findings were correlated with above.

**Results:**

Total of 72 patients were enrolled with mean age 61 years, mean BSA 1.9 m^2^, and left ventricular ejection fraction 45 ± 20%. Elevated RAP≥ 10 mmHg was associated with dilated IJV D_max_(1.0 vs. 0.7cm, p = 0.001), less RVD with resting respiration (14% vs. 40% for IJV, p = 0.001 and 24% vs. 45% for SCV, p = 0.001), and reduced likelihood of total collapsibility with sniff (16% vs. 66% patients for IJV, p = 0.001 and 25% vs. 57% patients for SCV, p = 0.01). For RAP ≥10 mmHg, lack of IJV complete collapsibility with sniff had a sensitivity of 84% while IJV D_max_ > 1cm and RVD <50% had a specificity of 80%.

**Conclusion:**

The IJV and SCV diameters and their respiratory variation are reliable in estimating RA pressure.

## Abbreviations

IJV: internal jugular vein
SCV: subclavian vein
D_max_: maximum diameter
D_min_: minimum diameter
RVD: respiratory variation in diameter
LVEF: left ventricular ejection fraction
RA: right atrial
ROC: receiver operating curve
IVC: inferior vena cava
CVP: central venous pressure
RV: right ventricular
PA: pulmonary artery
PCWP: pulmonary capillary wedge pressure
BMI: body mass index
LVAD: left ventricular assist device
AP: anteroposterior

## 1 Introduction

Assessment of a patient’s fluid status is a common daily assignment for clinicians, in both inpatient and outpatient settings. Accurate fluid-status evaluation is especially valuable in patients with heart failure, in order to adjust diuretic therapy, but also in patients with septic shock, pulmonary hypertension, etc. However, this estimation is often confounded by the body habitus, inaccurate daily input/output documentation and a disconnect between physical examination and actual intravascular status.

Recent technological advancements as well as development of hand-held devices have made ultrasound technology more compact, portable, widely available and inexpensive. This could change the “estimation” to direct “visualization” of fluid status. Inferior vena cava (IVC) imaging is mostly used for this assessment, however, this often requires special training and it is not always feasible due to patients’ body habitus among other reasons. Ultrasound of the internal jugular vein(IJV) or subclavian vein (SCV) is comparatively an easy skill to acquire relative to IVC assessment due to their superficial position. This is especially important at early stages in medical training of residents and for community hospitals lacking advanced equipment. During medicine rounds, IJV/SCV assessment can also save time compared to IVC assessment, increase patient comfort and provide an essential yet easy to adopt adjunct to daily fluid status monitoring of patients at bedside. Similarly, adoption of such practice is quite feasible in outpatient settings especially in specialty heart failure clinics.

Studies in the past have correlated IJV/SCV to IVC as the reference standard.^1^, ^2^, ^3^ However, these studies were inconclusive partly due to lack of invasive hemodynamic assessment for accurate validation of diagnostic performance.^4^ On the other hand, studies correlating IJV to direct invasively measured central venous pressure (CVP) were fraught with limitations of smaller non-uniform cohorts such as combining ventilated and non-ventilated patients.^5^

Accordingly, there have been no prospective studies correlating ultrasound assessment of IJV and SCV with invasive evaluation of right atrial pressure in non-ventilated patients. This study aims to provide a standardized technique for bedside ultrasound assessment of IJV and SCV, and correlate the results with invasive hemodynamics obtained immediately after. We hypothesized that the size and respiratory variation of IJV/SCV provides an accurate estimation of actual right atrial pressure measured through right heart catheterization.

## 2 Methods

From September 2018 to May 2019, patients were prospectively enrolled in the Jewish Hospital and University of Louisville Hospital, Kentucky. The study protocol was reviewed and approved by the institutional review board of the University of Louisville. All patients included in the study signed an informed consent. All patients scheduled to undergo right heart catheterization at the Jewish and University of Louisville Hospitals were eligible for the study. The inclusion criteria included: spontaneously breathing adults (age > 18 years) and able to consent. Patients with orthotopic heart transplant (OHT) or left ventricular assist device (LVAD) were also eligible for enrollment. Exclusion criteria included: known occlusion of IJV/SCV, superior vena cava obstruction/compression or severe tricuspid regurgitation.

For the purpose of the study, patients were educated about the study procedures including the sniff maneuver. Patients were then positioned supine at 0° with their head in neutral position and breathing restfully. Next, the right sternocleidomastoid muscle was identified and IJV was imaged at the apex of the triangle formed by the sternal and clavicular heads of the muscle. Similarly, the SCV was imaged at the junction of the lateral third and the middle third of the right clavicle. If the patient had an indwelling intravenous catheter or an implanted device, such as a pacemaker, on one side of the neck or chest wall then the contralateral vein was used.

**Fig. 1:**
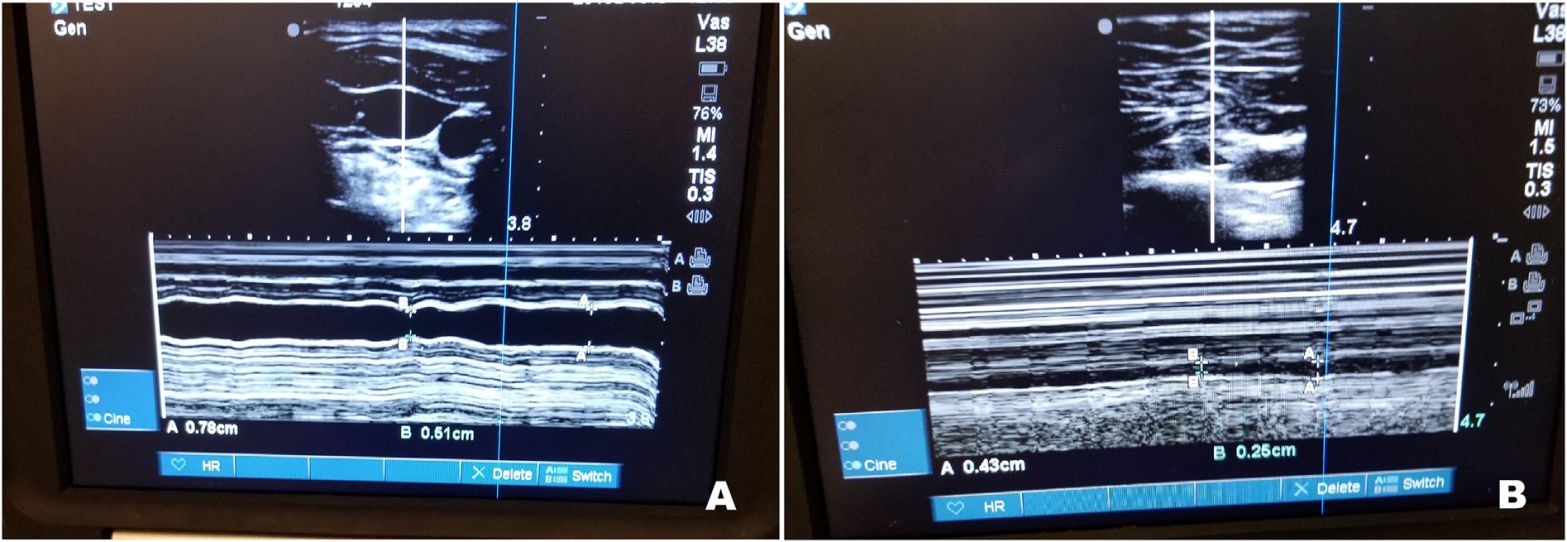
AM-mode ultrasound of the internal jugular vein, 1 B: M-mode ultrasound of the subclavian vein.

A portable ultrasound system- Sonosite (Bothell, WA) was used for this study. The ultrasound imaging acquisition was performed by a senior cardiology fellow (GV) to maintain uniformity. Using M-mode, the maximum and minimum anteroposterior diameters of IJV/SCV at the above-mentioned landmarks, were noted during normal breathing, without applying any external pressure (Fig. 1A and B). The respiratory variation percentage was calculated as [(maximum diameter – minimum diameter)/maximum diameter] and expressed as percent. The patients were then asked to sniff forcefully. The anteroposterior diameter collapsibility was assessed on sniff maneuver. The first 10 imaging acquisitions were timed.

The patients then underwent right heart catheterization within 1 h of the ultrasound assessment and right atrial (RA) pressure, right ventricular (RV) pressure, pulmonary artery (PA) pressure and pulmonary capillary wedge pressure (PCWP) were recorded.

### 2.1 Statistical analysis

IBM SPSS (version 24.0, SPSS Corp, Chicago, IL, USA) was used for statistical analysis. Qualitative data is presented as frequencies and quantitative data as mean ± standard deviation. Categorical variables and continuous variables were analyzed using Chi-square test, and Student’s *t*-test respectively. The correlation of imaging parameters to invasive RA pressure measurement was assessed using linear regression. Receiver operating curve (ROC) analysis was performed to determine the sensitivity and specificity of imaging parameters in estimation of right atrial pressure with the invasive RA pressure as the gold standard. A two-sided *p*-value <0.05 was considered significant.

## 3 Results

Total of 72 patients were enrolled in the study with mean age 61 ± 14 years, and mean BSA 1.9 ± 0.2 m^2^.None of the patients were ventilator dependent or on intravenous inotropic/vasoactive agents. Echocardiography data was available in 81% of patients within one month of enrollment and the mean LVEF was 45% (10–75%).Forty percent of patients had BMI ≥ 30 kg/m^2^(Table 1A).

**Table 1:** ABaseline characteristics of the patient population, 1 B: Right heart catheterization findings.

Variable	Frequency/Mean
Male	61%
Age (years)	60.8 ± 14.0 (21–85)
Body surface area (m^2^)	1.9 ± 0.2 (1.3–17.4)
Body mass index (kg/m^2^)	30.0 ± 6.5 (17.4–48.1)
Systolic blood pressure (mmHg)	125.4 ± 24.0 (54–196)
Heart rate (beats/min)	75.8 ± 15.5 (52–110)
Atrial fibrillation	9%
Trace/mild tricuspid regurgitation	89%
LV ejection fraction (%)	45.2 ± 20.0 (10–75)
Recurrent catheterization	14%
Serum Creatinine (mg/dL)	1.47 ± 1.46 (0.39–10.56)
Blood urea nitrogen/creatinine ratio	17.31 ± 6.6 (4.0–37.2)
Serum bicarbonate (mg/dL)	25.8 ± 3.5 (11.3–34.0)

Variable (mmHg)	Mean
Right atrial pressure (mmHg)	8.3 ± 5.3 (0–20)
Pulmonary systolic pressure (mmHg)	44.7 ± 20.2 (16–120)
Pulmonary mean pressure (mmHg)	28.8 ± 13.0 (10–74)
Pulmonary capillary wedge pressure (mmHg)	15.5 ± 9.3 (4–48)

Normal LVEF was defined as ≥ 52% in males and ≥54% in females based on American Society of Echocardiography guidelines.^6^ Half the patients with available data had normal ejection fraction and 42% had EF ≤ 35%. The cohort included 6 (8%) OHT recipients and 4 (6%) patient with LVAD implantation.

Image acquisition required <5 min per patient as assessed in the first 10 patients. IJV was imaged in all patients while SCV could not be imaged in 7 patients. These 7 patients had a BMI of 33.1 ± 7.6kg/m2 compared to others with a BMI of 29.6 ± 6.3kg/m2, p = 0.001.

Right heart catheterization findings are described in Table 1B; 35% of patients had RA pressure ≥10 mmHg and 31% had at least moderate pulmonary hypertension. All patients with maximum IJV diameter <0.5cm had RA pressure <10 mmHg (12 patients, 17%). Similar findings were noted with RVD in IJV >50% (16 patients, 22%).

Patients with elevated RA pressure (≥10 mmHg) showed less RVD in IJV with respiration in resting condition (14 vs. 40%, p = 0.01). They also had larger maximum IJV diameter (p = 0.01) [Table 2A]. Similar results were noted with SCV (Table 2B).

**Table 2:** AElevated RA pressure and IJV diameter variation on respiration, 2 B: Elevated RA pressure and SCV diameter variation on respiration.

	RA pressure ≥10 mmHg	RA pressure <10 mmHg	P-value
Maximum IJV diameter (cm)	1.0 ± 0.2	0.7 ± 0.3	0.001
Percent IJV diameter variation	14%	40%	0.001
Complete IJV AP collapsibility on sniff	16%	66%	0.001

	RA pressure ≥10 mmHg	RA pressure <10 mmHg	P-value
Maximum SCV diameter (cm)	0.8 ± 0.2	0.6 ± 0.3	0.270
Percent SCV diameter variation	24%	45%	0.011
Complete SCV AP collapsibility on sniff	25%	57%	0.012

Complete collapsibility of IJV anteroposterior diameter with sniff maneuver was associated with significantly lower RA (5.2 vs. 11.3 mmHg, p = 0.001) and PCWP pressures (12.2 vs. 18.5 mmHg, p = 0.004) [Table 3A]. Similarly, completely collapsible SCV had lower RA pressure (6.2 vs. 10.5 mmHg, p = 0.001) and PCWP (11.5 vs. 19.2 mmHg, p = 0.001)[Table 3B].

**Table 3:** AIJV collapsibility on sniff and the right heart catheterization findings, 3 B: SCV collapsibility on sniff and the right heart catheterization findings.

	IJV collapsible	Not collapsible	P-value
Right atrial pressure (mmHg)	5.2 ± 2.8	11.3 ± 5.4	0.001
Pulmonary systolic pressure (mmHg)	36.2 ± 12.7	52.7 ± 22.7	0.001
Pulmonary mean pressure (mmHg)	23.2 ± 8.2	34.1 ± 14.4	0.001
Pulmonary capillary wedge pressure (mmHg)	12.2 ± 7.3	18.5 ± 10.1	0.004

	SCV collapsible	Not collapsible	P-value
Right atrial pressure (mmHg)	6.2 ± 4.6	10.5 ± 5.4	0.001
Pulmonary systolic pressure (mmHg)	39.3 ± 19.3	49.9 ± 20.8	0.037
Pulmonary mean pressure (mmHg)	24.6 ± 11.2	32.7 ± 14.0	0.013
Pulmonary capillary wedge pressure (mmHg)	11.5 ± 6.1	19.2 ± 10.3	0.001

Sensitivity and specificity analysis were performed to assess accuracy of IJV/SCV ultrasound in estimating high RA pressure (Table 4). For RA pressure ≥10 mmHg, lack of IJV collapsibility with sniff had a sensitivity of 84% and specificity of 66%. A maximum IJV diameter ≥ 1cm and respiratory variation <50% had a sensitivity of 60% and specificity of 80% with ROC area 0.694 for RA pressure ≥10 mmHg (Table 4). Similarly, a maximum IJV diameter ≥ 1cm and lack of complete IJV collapsibility with sniff had a sensitivity and specificity of 56% and 83% respectively. The performance of SCV was not as robust, failing to reach significance with maximum diameter cutoff of 0.8cm (data not shown).

**Table 4:** Sensitivity and specificity of various IJV findings in predicting RA pressure ≥10 mmHg.

	Sensitivity	Specificity	ROC AUC
Maximum IJV diameter ≥1cm	60%	72%	0.662
No IJV collapsibility with sniff	84%	66%	0.750
Maximum IJV diameter ≥1cm + no collapsibility	56%	83%	0.708
Maximum IJV diameter ≥1cm + percent variation <50% on normal respiration	60%	80%	0.694

Among the subgroup of patients with EF ≤ 35% (30 patients), the percent diameter variation continued to have positive correlation with RA pressures (R = 0.66 for both IJV and SCV, p = 0.001). Similarly, for patients with mean pulmonary pressure ≥35 mmHg, the positive correlation between percent variation and RA pressure was maintained (R = 0.66 for IJV, p = 0.001 and R = 0.42 for SCV, p = 0.05). However, patients with recurrent catheterization (heart transplant and LVAD patients in the cohort) lost the positive correlation (R = 0.467, p = 0.174).

**Fig. 2:**
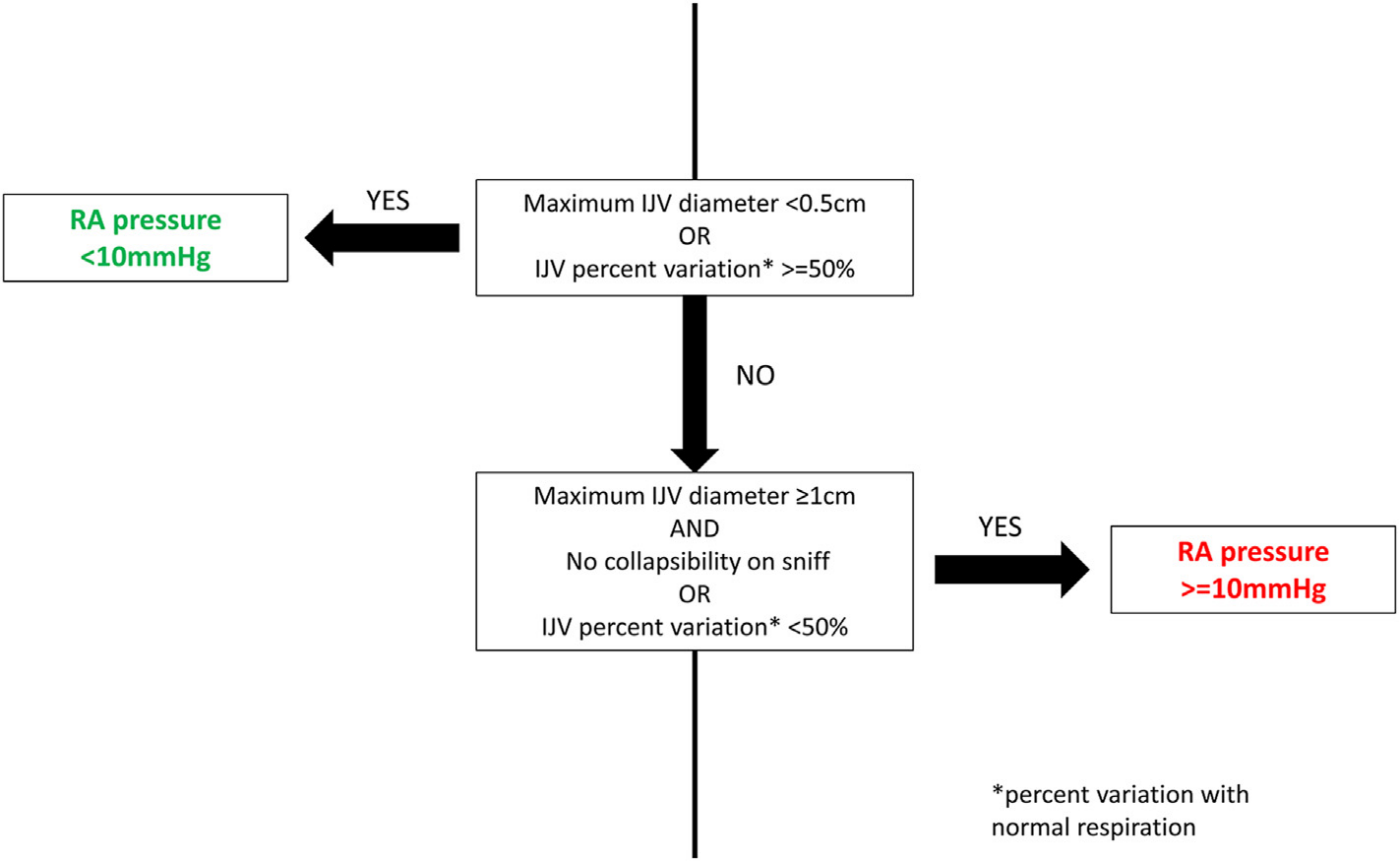
Algorithm for utilizing internal jugular vein diameter for estimation of right atrial pressure.

Based on the above data, an algorithm was constructed to estimate RA pressure at bedside with considerable certainty (Fig. 2).

## 4 Discussion

We report, for the first prospective correlation of both internal jugular and subclavian vein diameters as well as their collapsibility, as assessed with bedside ultrasound, with invasive right heart catheterization. The study cohort represents a real-world population of patients including patients with heart failure, pulmonary hypertension, LVAD and heart transplant. We demonstrated 1) A significant positive correlation between the vein diameters and RA pressure, 2) Less respiratory variation and larger vein diameters with elevated RA pressure, and 3) Lack of IJV collapsibility as a highly sensitive marker for higher RA pressure.

The study results have direct application in day-to-day care of patients with actual or suspected heart failure in addition to patients with sepsis, pulmonary hypertension, etc. Accurate estimation of fluid status is of paramount importance in patients with heart failure, for guidance in treatment as well as monitoring response to diuretic therapy. However, this estimation is often confounded by the body habitus, inaccurate daily fluid intake/output documentation and a disconnect between physical examination and actual intravascular fluid status. Frequent invasive right heart catheterization is neither feasible nor desirable.^7^ Under such circumstances, direct estimation of intravascular volume status is often sought through visualization of inferior vena cava (IVC) diameter as well as respiratory variation.^8^, ^9^ Alternative systemic veins available for this purpose include IJV and SCV.

Ultrasound of IJV or SCV is an easy skill to acquire as compared to IVC assessment due to their superficial position. This is especially important at early stages in medical training of residents and for community generalists lacking advanced equipment or skills. During medicine rounds, IJV/SCV assessment can be an essential yet easy to adopt adjunct to daily fluid status monitoring of patients at bedside.

There have been studies correlating IJV and SCV to IVC.^1^, ^2^, ^3^ However, using IVC as gold standard is fraught with inaccuracies resulting from improper/inadequate imaging technique and non-standardized patient positioning as well as confounded by intra-abdominal pressure and body habitus.^4^ As expected, these studies did not have encouraging results. Moreover, IVC evaluation has been shown to be unreliable in predicting fluid responsiveness.^8^ In this setting, there have been no studies correlating ultrasound assessment of both IJV and SCV with invasive catheter-directed hemodynamic evaluation.

Studies performed in the past addressing IJV to invasive CVP correlation have shown mixed results, using various IJV measurement parameters. In their study on critically ill patients, Avcil et al^5^ reported a complex relationship between IJV and CVP, with maximum IJV diameter performing best in patients with low CVP (<6 mmHg). The caveat is that they enrolled both mechanically ventilated and spontaneously breathing patients. Mechanical ventilation has a fundamental effect on patients’ hemodynamics and therefore interpretation of their findings is difficult. Killu et al^10^ similarly reported that an IJV RVD >39% was a reliable marker of hypovolemia. However, the study was restricted to detecting hypovolemia in surgical critical care patients and not hypervolemia in heart failure patients.

On the other hand, in a small cohort of 34 patients, including only 7 patients with central venous pressure >10 cm of water, Donahue et al^11^ reported positive correlation between CVP and IJV diameter. Another study on mechanically ventilated patients noted a good predictive value between high/low CVP with an IJV diameter ratio obtained both at 0° and 30° tilt.^12^ Prekker et al^13^ reported that IJV height-to-width ratio at end-expiration performed poorly compared to maximum IVC diameter in estimating RA pressure. Siva et al^14^ estimated the height of IJV pulse which correlated well with CVP measurement. Simon et al^15^ reported that respiratory variation in IJV cross-sectional area traced manually along the inner-edge predicted the RA pressure accurately.

The present study used IJV and SCV measurements to mimic the current mainstream IVC quantification: maximum diameter, respiratory variation and sniff maneuver. We only recruited spontaneously breathing patients to avoid confounding effects of mechanical ventilation. The ultrasound measurements were uniformly performed in all patients. Notably, the invasive hemodynamic assessment was performed within 1 h of the ultrasound measurements reducing the likelihood of a major shift in patients’ hemodynamic status.

We decided to focus on the discriminative value of IJV and SCV parameters in estimation of higher RA pressures which is of paramount importance in the treatment of patients with heart failure. There was less respiratory variation in IJV/SCV diameters with elevated RA pressures and a lack of IJV collapsibility was a highly sensitive marker for higher RA pressure (84% sensitivity). A maximum IJV diameter ≥ 1cm and respiratory variation <50% improved the specificity to 80% for RA pressure ≥10 mmHg. An algorithm was devised based on the above findings (Fig. 2).

In studies correlating IVC to invasive hemodynamics, a preference was given to detecting low CVP using IVC imaging.^5^ An IVC diameter < 2cm had a sensitivity ranging from 73 to 85% and specificity of 81–85% for CVP <10 mmHg.^13^, ^16^ Similarly, an IVC RVD of >40–50% had a sensitivity ranging from 73 to 91% and specificity ranging from 84 to 94% for detecting CVP <10 mmHg.^16^, ^17^ The performance of IJV in our study closely mimics the performance of IVC, albeit for higher CVP (Table 4).The correlation persisted in patients with EF ≤ 35% (R = 0.66 for both IJV and SCV).

Patients with recurrent catheterization did not have a positive correlation between percent diameter variation and RA pressure. Such patients were either heart transplant recipients (6 patients) or had undergone LVAD implantation (4 patients). These were also the only patients who had undergone an open-heart surgery. This could be due to fibrotic changes/partial occlusion in the veins related to recurrent instrumentation, lack of physiological variation due to mechanical device/venous grafting or from scarring around superior vena cava from open-heart surgery.

The study had a number of limitations. Although the sample size was larger than other studies comparing vein measurements with invasive hemodynamics, the findings will need validation in larger studies for wider application. The study did not involve mechanically ventilated patients which can affect the central venous size independent of the CVP. The study was also underpowered to examine volume status of patients in a more granular fashion, but instead focused on a binary distribution. Since we had invasive RA pressure measurement for all patients, imaging of IVC or clinical assessment of the jugular venous pulse were not performed. Moreover, we did not aim to compare various noninvasive modes of fluid status assessment but to demonstrate the feasibility and accuracy of IJV/SCV assessment at bedside. Finally, the ultrasound imaging was performed by a single operator and inter-observer variability could not be assessed.

## 5 Conclusion

The IJV and SCV diameters are reliable and easier alternatives in bedside estimation of RA pressure.

## Compliance of ethical standard

Statement of human rights: All procedures performed in studies involving human participants were in accordance with the ethical standards of the institutional and national research committee and with the 1964 Helsinki declaration and its later amendments or comparable ethical standards.Informed consent: Informed consent was obtained from all individual participants included in the study.

## Disclosures

No financial disclosures from any of the authors. No conflict of interest to declare.

## Declaration of competing interest

The authors declare that they have no conflict of interest.
